# SLNP-based CDK4- targeted nanotherapy against glioblastoma

**DOI:** 10.3389/fonc.2024.1455816

**Published:** 2024-11-22

**Authors:** Uzma Ghani, Fareeha Khalid Ghori, Muhammad Usman Qamar, Hina Khan, Basit Azad, Sabahat Habib, Saira Justin, Ishaq N. Khan, Tawaf Ali Shah, Gamal A. Shazly, Mohammed Bourhia, Fouzia Perveen, Aneela Javed

**Affiliations:** ^1^ Molecular Immunology Laboratory, Department of Healthcare Biotechnology, Atta-ur-Rahman School of Applied Biosciences, National University of Sciences and Technology, Islamabad, Pakistan; ^2^ Institute of Microbiology, Faculty of Life Sciences, Government College University Faisalabad, Faisalabad, Pakistan; ^3^ Division of Infectious Disease and Department of Medicine, University of Geneva, Geneva, Switzerland; ^4^ Materials And Modeling Lab, School of Interdisciplinary Engineering and Sciences, National University of Sciences and Technology, Islamabad, Pakistan; ^5^ Cancer Cell Culture and Precision Oncomedicine Lab, Institute of Basic Medical Sciences (IBMS), Khyber Medical University, Peshawar, Pakistan; ^6^ Department of Pharmaceutical Sciences, Taxes A&M Health Science Center, Joe H. Reynolds Medical Sciences Build, College Station, TX, United States; ^7^ College of Agriculture Engineering and Food Sciences, Shandong University of Technology, Zibo, China; ^8^ Department of Pharmaceutics, College of Pharmacy, King Saud University, Riyadh, Saudi Arabia; ^9^ Department of Chemistry and Biochemistry, Faculty of Medicine and Pharmacy, Ibn Zohr University, Laayoune, Morocco

**Keywords:** blood brain barrier, glioblastoma, silymarin, solid lipid nanoparticles, temozolamide

## Abstract

**Introduction:**

Glioblastoma is a grade IV solid brain tumor and has a 15-month survival rate even after treatment. Glioblastoma development is heavily influenced by retinoblastoma protein (pRB) pathway changes. The blood–brain barrier, drug resistance, and severe toxicity of Temozolamide are key obstacles in treating glioblastoma. Innovative treatments targeting the pRB pathway with efficient delivery vehicles are required to treat glioblastoma.

**Methods:**

For this purpose, a library of 691 plant extracts previously tested *in vitro* for anti-cancerous, anti inflammatory, and anti-proliferative characteristics was created after thorough literature investigations. Compounds were docked against pRB pathway protein ligands using molecular operating environment and chimera. Their nuclear structure and drug-like properties were predicted through Lipinski rule and density functional theory analysis. Physio-chemical characterizations of naked and drug-encapsulated SLNPs assessed size, stability, entrapment efficiency, and drug release rate. Anti-cancer potential of drug and drug- loaded SLNPs was evaluated using U87, U251, and HEK cell lines. Formulations were tested for cancer cell metastatic potential using cell migration assays.

**Results:**

Silymarin (Sil) was identified as the most potent compound against CDK4, which was then encapsulated in stearic acid solid lipid nanoparticles (SLNP-Sil). Sil showed decreased cell viability 72 h after treatment against both U87 and U251 cell lines but had negligible cytotoxic effect on HEK-293. IC50 value of Sil was 155.14 µM for U87 and 195.93 µM for U251. Sil and SLNP-Sil effectively inhibited U87 and U251 cell migration 24 h after treatment.

**Discussion:**

Our results indicated that Sil and SLNP-Sil are promising therapeutic approaches against glioblastoma and merit *in vivo* experimental verification using orthotropic xenograft mouse models against glioblastoma.

## Introduction

Glioblastoma is the most aggressive, invasive, and common type of primary brain tumor, with over 17,000 new cases recorded each year in the United States (1). Despite rigorous multimodal treatment, the overall survival duration following diagnosis of glioblastoma is reported to be fewer than 15 months and less than 5% of patients still alive at 5 years (2–4). Genomic profiling and The Cancer Genome Atlas project revealed that alteration in three core signaling pathways, namely, P53, PI3K, and pRB, greatly contributes to the development of glioblastoma (5–7).

Dysregulation of the pRB pathway disrupts normal cell-cycle control, allowing tumor cells to bypass key checkpoints that usually prevent unchecked cell division. This leads to uncontrolled proliferation and increased cell survival, a common feature in many cancers, including glioblastomas (8). The cell cycle is tightly regulated by pRB pathway proteins (CDKs), whose activity is crucial for progressing through different phases of the cell cycle. In glioblastoma, overexpression of CDK2, CDK4, and CDK6 is frequently observed, underscoring their critical role in driving astrocytic tumorigenesis and glioma progression (9). High levels of CDK expression not only correlate with increased tumor proliferation but are also negatively associated with glioblastoma patient survival. Given their central role in cell-cycle regulation, these CDKs are promising targets for therapeutic strategies in glioblastoma (10).

Temozolomide (TMZ) is currently the standard drug used for the treatment of glioblastoma. However, 50% of patients with glioblastoma have been non-responsive to the drug due to elevated O6-methylguanine methyltransferase expression (11, 12). Additionally, *in vitro* studies have shown a substantial increase in the number of glioblastoma stem cells within the total glioblastoma cell population following treatment with TMZ (4). Furthermore, TMZ use is frequently linked to severe dose-related toxicity and an elevated risk of bone marrow suppression (11, 12), thus necessitating the development of new alternate therapy options against patients with glioblastoma to improve glioma prognosis and survival by targeting the key pathways and proteins implicated in glioblastoma development (13).

Plants contain naturally occurring secondary metabolites that are being studied for anti-cancer properties, with the goal of developing novel therapeutic medications (14, 15). They have a wide range of phytochemicals that can target aggressive brain tumors and induce apoptosis (16). Presence of blood– brain barrier (BBB) is another obstacle which prevents chemotherapeutic drugs to reach the target site and perform their function efficiently (17). Plants are the primary source for the treatment of a plethora of diseases both in traditional medicines and contemporary herbalism procedures. Medicinal plants constitute a reservoir of natural products providing new molecules with anti-cancer potentials and providing basis for the design of derivatives with improved therapeutic ability (18). Additionally, plant-derived compounds are less or non- cytotoxic to normal cells, representing a source of anti-cancer molecules with lesser side effects as compared to current chemotherapeutic/synthetic drugs (15).

The use of nanotechnology in targeted drug delivery systems including of nanoparticles (NPs) and nanostructured materials improved the delivery and local concentration of drugs owing to their optimal size as well as drug- loading and drug-releasing characteristics, and crossing BBB improved drug distribution and their enhanced circulation in blood stream. Various studies decipher the role of nanodelivery systems as a presumptive power tool for the delivery of both single and multimodal medicines (19). Solid lipid nanoparticles (SLNPs) carry both hydrophobic as well as hydrophilic drug. SLNPs combine the beneficial qualities and avoid the drawbacks of several nano-carries including emulsions, liposomes, and lipospheres (20). Coating excipients such as polysorbate-80 (P80) allows effective penetration across BBB. Due to the unique ability of P80 to mimic the nano-carrier as low-density lipoprotein (LDL), it is identified as its own ligand by the BBB’s LDL receptors and taken up by endocytosis (21).

The conventional method of discovering a new drug for any disease is time- consuming and costly. In recent years, the anticipated cost of bringing a new medicine to market has risen to almost $1.8 billion USD, with a 96% attrition rate for drug candidates. Computer-aided drug discovery (CADD) approaches have recently gotten a lot of interest because they can help with the scale, time, and cost issues of traditional experimental approaches. Computational drug target discovery, virtual screening of huge chemical libraries for viable drug candidates, further optimization of selected compounds, and *in silico* toxicity assessment are all part of CADD, followed by *in vitro/in vivo* testing (22). Various powerful tools are currently being employed for this aim. Current study has utilized the techniques and identified Silymarin (Sil) from a pool of potential anti-cancer drugs against glioblastoma based on the predicted results. Sil was further conjugated into SLNPs, and, after thorough characterization, both naked and nanoconjugate drugs were tested *in vitro* using glioblastoma- specific cell lines, i.e., U87-MG and U251-MG, as well as non-cancerous cell line HEK-293 as control; TMZ (a standard anti-cancer drug) was used as a positive control drug for comparative analysis.

## Materials and methodology

### *In silico* methodology

#### Preparation of database

A 10-year literature survey (2010–2020) for plant-based chemicals with anti-inflammatory, anti-proliferative, and anti-cancer activities along with the tested cancer types was carried out. A total of 691 plant-based compounds possessing the above properties were collected, and PubChem was accessed to retrieve their Simplified Molecular Input Line Entry System (SMILES). A simple dataset having the common or scientific names of the compounds, their SMILES, and cancer types was created. By using SMILES, a database of these 691 compounds was created by and saved for further screening and molecular docking investigation.

#### Ligand and protein preparation for docking

To prepare the ligands for docking, their energies were minimized using the default parameter prior to saving as database. The target proteins with Protein Data Bank (PDB) IDs 6GUH, 3G33, and 4AUA and resolutions of 1.50, 3.00, and 2.20 were retrieved from the Research Collaboratory for Structural Bioinformatics (RCSB) PDB (23–25). Structures of proteins were purified by removing all bound ligands and water molecules using the chimeraX1.1 program. Their macromolecular structures were relaxed by energy minimization through Molecular Mechanics (MM) method using MOE Chemical Computing Inc. (2018) and were saved for further molecular docking pattern with the ligands.

#### Molecular docking

To perform molecular docking studies, crystal structures of the proteins were minimized by using Amber10 force field and were taken as receptors. A database of the 691 compounds was also prepared by refining the chemical correctness (3D protonation), ionization, and stereochemical variation and was imported for docking simulations as ligand. The resulting models of protein were subjected to systematic conformational search at default parameters with RMS gradient of 0.01 kcal mol^−1^ using Site Finder (26, 27). A 100 calculation runs were performed in order to get a final binding pose of docked structures as accurate as possible. The best conformation was screened on the basis of energetic ground, and best docking pose was selected on the basis of minimum scoring function (R) (27, 28).

#### Lipinski rule

The top compounds obtained after docking, with lowest S score were further screened by applying the Lipinski rule of drug likeness to choose a compound that is targeting multiple cell regulatory proteins used in this study. For this, the SwissADME program (29) was employed. Screening of the compounds through the Lipinski rule, targeting CDK4 protein, was also performed.

The screening criterion selects only compounds that meet the following conditions:

Having ≤ 5 hydrogen bond donors: Molecules with too many hydrogen bond donors may be too polar, which can hinder their ability to cross cell membranes and be absorbed (30).

Having ≤ 10 hydrogen bond acceptors: Excessive hydrogen bond acceptors can make a molecule too polar, reducing its ability to pass through lipid-rich environments like cell membranes (31).

Molecular mass of ≤ 500 Daltons: Larger molecules may have difficulty crossing cell membranes and are more likely to be metabolized or excreted before reaching their target (32).

Log P of ≤ 5: Compounds with a high Log P are too lipophilic (fat-loving) and may accumulate in fatty tissues or have poor solubility in water, affecting absorption and distribution (30).

Fewer than 10 rotatable bonds: Too many rotatable bonds can make a molecule too flexible, reducing its ability to bind effectively to its target (33).

#### Quantum chemical studies

To select the best compound targeting our desired protein, discrete quantum chemical studies were carried out using AMS-Application Development Framework (ADF) 2022. ADF builder was used to generate structures and visualize graphics for the compunds (34). Top five compounds were selected on the basis of best scoring function from molecular docking: Sil, Omtriptolide, Belamide, isoMalygamide, and Daphnoretin were optimized by first- principle density functional theory (DFT) calculations performed at generalized gradient approximation (GGA) of Perdew-Burke-Ernzerhof (PBE) functional with TDZ basis set (35). Frontier molecular orbitals, molecular electrostatic potential, and band- gap analysis were also performed at GGA: PBE/DZ level of theory. These parameters provided insight into the reactivity of compounds.

### *In vitro* anti-cancer potential analysis

#### Materials

Materials used are as follows: Dulbecco's Modified Eagle Medium (DMEM) (1×) from SolaBio, 10% fetal bovine serum (FBS) from Gibco, Pen-Strep from Gibco, trypsin from Sigma, 0.4% trypan blue from Gibco, reagent- grade stearic acid (95%) from Sigma, polysorbate-80 from Sigma, phosphate-buffered saline (PBS) from Invitrogen, Dimethyl sulfoxide (DMSO) from Gibco, Sil (80% purity) from MACKLIN, TMZ from Eirgen Pharma, and methanol from Honeywell.

#### Methods

##### Preparation of SLNPs

Drug-loaded SLNPs were prepared using the solvent injection method. For the organic phase, 20 mL of isopropanol was used to dissolve 380 mg of stearic acid, 200 mg of lecithin, and 100 mg of powdered Sil. The organic phase mixture was heated to 75°C and continuously stirred with a medium-sized magnet at 700 rpm for 60 min. The aqueous phase was prepared by stirring 1 g of polysorbate 80 in 60 mL of PBS for 40 min at 700 rpm and 75°C. Cooled distilled water (15 mL) was added after injecting 6 ml mL of the organic phase into the aqueous phase. After 2. 5 h of mixing, the dispersion was transferred into falcons, passed through a 0.2-m filter, and centrifuged at 7,500 for an hour at 4°C. The pellets were dried with a freeze-drier after the supernatant was discarded. The same process was used to prepare blank SLNPs (36, 37).

##### Characterization of SLNPs

###### Scanning electron microscopy

Scanning electron microscopy (SEM) analysis was performed to examine size and morphology of SLNPs. Blank (1 mg) and drug- incorporated (1 mg) SLNPs were weighed and dissolved in 2 mL of PBS solution. For 15 min, the solution was maintained at 37°C in an incubator and thoroughly sonicated until it became clear. One drop of the clear solution was placed on a piece of glass and dried in an incubator at 50°C. Before SEM analysis, the samples were coated with gold particles using an ion sputter. Samples were inspected at various resolutions during the study, and images were taken with the size (in nm) written on them (36, 38).

###### Fourier transform infrared spectroscopy

To validate the encapsulation of drugs into SLNPs, Fourier transform infrared spectroscopy (FTIR) analysis was performed. FTIR spectra of SLNPs, drugs, and drug-infused SLNPs were recorded using Nicolet Magna-IR 560 optical bench (Madison, WI, USA). The KBr disc method was used to prepare the samples for FTIR, which involves pressing KBr and sample with 5 Kpsi pressure to prepare a disc. The pressed samples were carefully removed and evaluated in an FTIR system at wave numbers ranging from 4,000 cm^−1^ to 400 cm^−1^ (39).

###### X-ray diffraction analysis

X-ray diffraction (XRD) analysis of SLNPs, drugs, and drug- infused SLNPs was performed to study the crystallinity of drugs when they are loaded in SLNPs. This was performed by placing the samples in sample disc of 0.5 mm thickness, and the sample disc was placed in disc holder. A monochromatic, collimated X-ray beam was passed through the samples, discharged from a Rigaku-Denki RU3 rotating copper anode generator. A one-dimensional quartz wire detector was used to record diffraction patterns for about 1 h, ranging from 0° to 70° (40).

##### UV/Vis spectrophotometry

###### Calibration curve of Sil

Calibration cure of the Sil was drawn through UV/Vis spectrophotometry method, which is the most critical step to evaluate drug entrapment and release efficiency. The drug (15 mg) was measured and dissolved in 2 ml mL of methanol through sonication for 2–5 min. This solution was diluted to 15 ml mL of methanol to get the stock solution having final concentration of 1 mg/mL. Dilutions of 100 µl/mL, 400 µl/mL, 500 µl/mL, 800 µl/mL, and 900 µl/mL were further prepared from the stock, 3 mL of each dilution was placed in the sample holder after blank, and absorbance values of all these dilutions were taken at lambda max, reported in the literature for Sil. A graph (calibration curve) was drawn with these absorbance values in Excel, and the equation y = mx + c was generated. The calibration curve generated was used to find the amount of entrapped and un-entrapped drug in SLNPs (41).

###### Drug entrapment efficiency of SLNPs

The percentage of Sil encapsulated in SLNPS was determined in terms of entrapment efficiency. SLNP-Sil final suspension was centrifuged for an hour at 700 rpm. Supernatant was collected and filtered through 0.2-µm syringe filter. The supernatant (300 µL) was suspended in 2,700 µL of methanol, and the absorbance value of un-entrapped drug was measured at lambda max of Sil. This value was put in the y = mx + c equation generated through calibration curve generated from Sil drug absorbance to find the amount of un-entrapped drug, and the entrapped drug %age was calculated by the formula mentioned below:


$$\begin{array}{c} \;\text{Entrapment Efficiency }\left(\%\right)\\ \;=\;\frac{Total\;amount\;of\;drug-free\;unentrapped\;drug\;in\;suspension}{Total\;amount\;of\;drug}\;\times 100 \end{array}$$

(36, 42)

###### Drug release efficiency of SLNPs

Drug release efficiency of SLNP-Sil was calculated through UV spectrophotometer. The freeze- dried SLNP-Sil (15 mg) was weighted and dissolved in 15 ml mL of methanol to make 1 mg/mL, equalent to 1,000 µM (482.44 µg) of Sil. The suspension was placed on the shaker and incubated at 37°C. ml The suspension (3 mL) was taken at specific time intervals (15 min, 30 min, 1 h, 6 h, 12 h, and 24 h). Equal volume of the solvent was added to the suspension after every removal to maintain sink condition. Each of the samples was centrifuged at 7,000 rpm for 30 min, and concentration of Sil in the supernatant at each time interval was calculated through finding the absorbance and putting the absorbance values in straight line equation obtained from the calibration curve of Sil. The concentration obtained was converted into %age by the formula given below:


$$\%\text{age Sil released }=\frac{\;\text{Amountof Sil obtained}}{Total\;amount\;of\;drug\;in\;1mg\;SLNPs}\;\times 100$$

(43)

##### Evaluation of anti-cancer potential

###### Culturing of GBM and normal cell lines

U87-MG (RRID: CVCL_0022), U251-MG (RRID: CVCL_0021), and HEK-293 (RRID: CVCL_0045) adherent cell lines were used in cell cytotoxicity experiments. T-25 flasks with pre-warmed full DMEM were used to cultivate the cells. To prevent contamination of microorganisms, 10% FBS and 1% Pen-Strep were added to the culture medium. To enable cell proliferation, the culture flasks were housed in a CO_2_ incubator at 37°C. To avoid contact inhibition, the cells were kept at a density of less than 1.0 × 10^5^/mL (75%).

###### MTT assay

MTT [3-(4, 5-dimethylthiazol-2-yl)-2, 5- diphenyltetrazolium bromide] assay was utilized to observe the anti-tumor potential of drugs by calculating % cell viability (44). On the first day, U87, U251, and HEK-293 cells were seeded in 96- well plates with a density of 1.0 × 10^4^ cells per well, and 100 µL of medium with cells was added to each well. Plates were placed in the incubator for 24 h, so that the cells could adhere to the surface of the well. After 24 h, Sil, TMZ drug, blank SLNPs, SLNP-Sil, and SLNP-TMZ dilutions were added in triplicates and left for further 24 h, 48 h, and 72 h to check the effect of these formulations on cells. On days 3, 4, and 5, 15 ml mL of MTT dissolved in PBS (5 mg/mL) was added to each well after 24 h, 48 h, and 72 h had passed. For the reduction reaction to occur, the plates were incubated for another 3 h. The wells were gently drained using pipetting after a 3-h incubation period with MTT. To precipitate the purple-colored insoluble crystals of formazan that were entrapped in the live cells, 100 µL of sterile DMSO was further added to the wells and incubated for further 30 min at 37°C. The absorbance of formazan in each well was detected at 550 nm, through spectrophotometer micro plate reader (36). Percentage cytotoxicity was calculated as follows:


%Cell viability=As/Ac×100

where As and Ac represent the absorbance value of the sample and control, respectively.

### Statistical analysis

To perform statistical analysis GraphPad Prism was utilized. All of the groups were compared using a two-way ANOVA to see whether there were any statistically significant differences between them. A p-value threshold of less than 0.05 was used to evaluate statistical significance, and the data were reported as mean + standard deviation (SD). To ensure the accuracy and clarity of our data, graphs were created using GraphPad Prism to visually display the cytotoxicity patterns and emphasize important variations between experimental settings.

### Cell migration assay

Cell scratch assay was performed on U87 and U251 cell lines to observe cell migration. The cells were seeded in 96- well plates and placed in the incubator for 24 h to form a monolayer with approximately 80% confluency. The wells containing monolayer cells were scratched the next day with sterile tip of 200 µL followed by a PBS washing to remove debris, and fresh medium was added to the cells. Cells were then treated with the highest concentrations of the samples used in MTT. After another 24 h of incubation, the plates were examined under microscope, and images were taken to observe cell migration (45). Quantitative analysis of cell migration rate was performed through ImageJ software that measures area uncovered by the cells. Values obtained were put in the formula given below to calculate percentage of wound healing.


$$\%\text{Wound healing}=\;\left({\text{At}}_{0}-{\text{At}}_{24}/{\text{At}}_{0}\right)\times 100$$

where At0 represents wound area measured directly after scratch and At24 represents wound area measured 24 h after scratching (46).

## Results

### Docking of plant compounds with GBM proteins

In the present study, a database of 691 plant compounds was created, and their molecular interactions were studied against RB pathway proteins, namely, CDK2, CDK4, and CDK6. The interaction behavior was monitored, and, through pose view analysis, binding strength was determined as per the binding affinity energies. The optimal interaction pose was determined by the conformation with the lowest binding energy value. The most potent compound targeting CDK2 and CDK6 was found to be Oenothein B, whereas Charantin against CDK4 was found to be most potent. The top 10 compounds having the most negative binding values for each targeted protein are shown in **Table 1**.

**Table 1:** Binding affinity energies (in kcal/mol) against CDK2, CDK4, and CDK6.

Sr. no.	Binding energies kcal/mol (CDK2)	Compound	Binding energies kcal/mol (CDK4)	Compound	Binding energies kcal/mol (CDK6)	Compound
1	−10.0026	Oenothein B	−9.1669	Charantin	−12.1011	Oenothein B
2	−9.3546	Charantin	−7.9509	Kraft lignins	−10.2415	Bleomycin
3	−8.651	Kraft lignins	−7.7859	Lyngbyastatin 9	−9.7796	Rutin
4	−8.2195	Thapsigargin	−7.7375	chebulinic acid	−9.6363	Linariin
5	−8.121	chebulinic acid	−7.7199	Hectochlorin	−9.2478	Ardisiacrispin B
6	−8.0192	Tasiamide B	−7.5889	Wewakazole	−9.2003	Pelargonidin-3,5-diglucoside
7	−7.9021	Tannin	−7.5364	Silymarin	−9.0735	Hyperoside
8	−7.8482	Rutin	−7.4418	Largamide G	−8.7403	chebulinic acid
9	−7.7442	Tannin	−7.4329	Tannin	−8.5562	Cyanidin-3-O-glucoside
10	−7.7406	TIC10	−7.4032	Coibamide	−8.3712	Lyngbyastatin 4

### Compounds targeting multiple proteins

The compounds targeting multiple proteins and having the highest binging energies were further screened through the Lipinski rule to find out whether any of these common compounds can be used as anti- glioblastoma drug and can further be tested *in vitro*. None of these compounds were found to be following the Lipinski rule, hence concluding that they cannot be used as multi-target drug (**Table 2**).

**Table 2:** Top compounds targeting multiple proteins (CDK2, CDK4, and CDK6).

Sr. no.	Common compounds	CDK2	CDK4	CDK6	Lipinski rule	Violations
1	Chebulinic acid	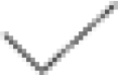	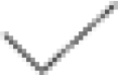	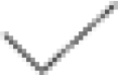	No; 5 violations	Mass > 500 NorO > 10 NHorOH > 5 LOGP > 5 17.467085 Molar Refractivity > 130
2	Oenothein B	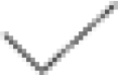		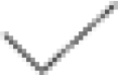	No, 4 violations	Mass > 500 NorO > 10 NHorOH > 5 Molar Refractivity > 130
3	Kraft lignin	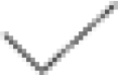	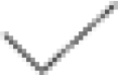		No, 4 violations	Mass > 500 NorO > 10 NHorOH > 5 Molar Refractivity > 130
4	Charantin	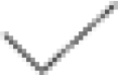	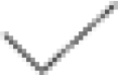		No; 3 violations	Mass > 500 NorO > 10 NHorOH > 5
5	Tannin	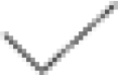	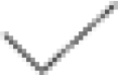		No; 3 violations	Mass > 500 NorO >10 NHorOH > 5

As none of the top compounds were found to act as a multi-target drug, targeting all the proteins (**Table 2**), compounds targeting only CDK4 that is primarily downregulated in the chosen GMB pathway were further screened through the Lipinski rule to identify the final five compounds that can be further screened through DFT analysis (**Table 3**).

**Table 3:** Top five compounds against CDK4, following the Lipinski rule.

Sr. no.	Compounds	Binding score	Number of violations
1	Silymarin	3.4 × 10^5^	Yes; 0 violation
2	Omtriptolid	97,396.01085	Yes; 0 violation
3	Belamide A	94,432.10648	Yes; 1 violation: MW > 500
4	Isomalyngamide A	84,672.24558	Yes; 1 violation: MW > 500
5	Daphnoretin	60,812.34714	Yes; 0 violation

### DFT analysis

#### Structural analysis

**Figure 1:**
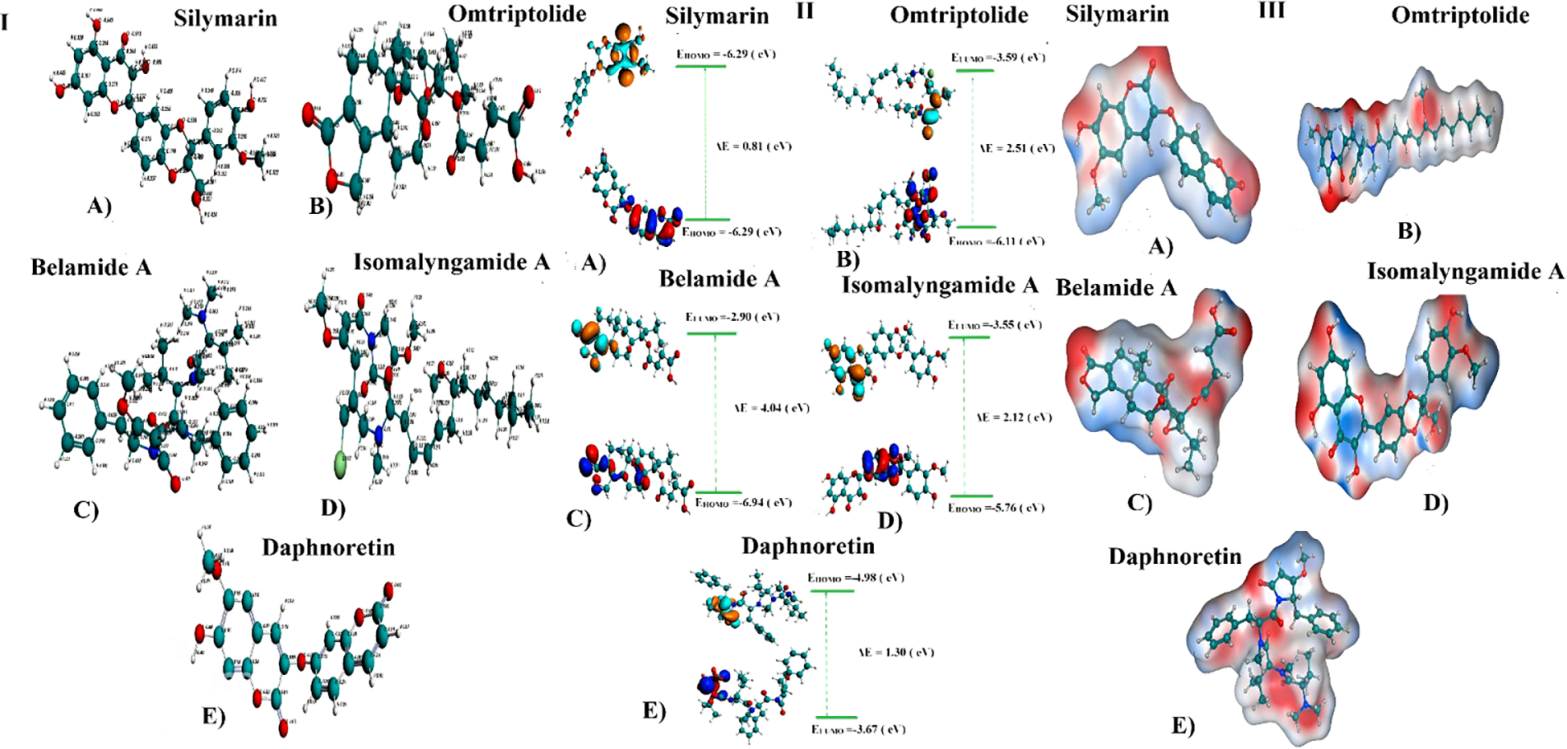
**(I)** Optimized geometries and charge distribution on **(A)** Silymarin, **(B)** Omtriptolid, **(C)** Belamide A, **(D)** Isomalyngamide A, and **(E)** Daphnoretin. **(II)** HOMO, LUMO, E_HOMO_, E_LUMO_, and band gap (ΔE) for **(A)** Silymarin, **(B)** Omtriptolid, **(C)** Belamide A, **(D)** Isomalyngamide A, and **(E)** Daphnoretin. **(III)** Molecular electrostatic potential surfaces (MESPs) of **(A)** Silymarin, **(B)** Omtriptolid, **(C)** Belamide A, **(D)** Isomalyngamide A, and **(E)** Daphnoretin.

DFT/GGA: PBE was used to model and optimize structural geometries of Sil, Omtriptolid, Belamide A, Isomalyngamide A, and Daphnoretin to determine geometric (bond lengths and bond angles) and electronic characteristics (E_HOMO_, E_LUMO,_ and ΔE). The optimized geometries of Sil, Omtriptolid, Belamide A, Isomalyngamide A, and Daphnoretinas as well as the symmetrical charge distribution on each individual atom of these compounds are shown in **Figure 1 (I)**.

#### Frontier molecular orbital analysis

The molecular orbital frontier analysis was performed using a quantum mechanical approach, which is considered as a most popular tool to anticipate chemical transitions (47, 48). The E_HOMO_ and E_LUMO_ values offer an idea of the nature of an electron-donating or electron-accepting compound, and, thus, a compound is deemed to be more electron-donating when value of its E_HOMO_ increases and more electron-accepting when the value of its E_LUMO_ decreases (49). The corresponding E_HOMO_, E_LUMO_, and ΔE values for each of the compound are shown in **Figure 1 (II)**.

The values in **Figure 1** revealed that the electron transfer in Sil is more viable as compared to Omtriptolid, Belamide A, Isomalyngamide A, and Daphnoretindue to smaller highest occupied molecular orbital (HOMO)-Lowest unoccupied molecular orbital (LUMO) gap. To understand this, the isodensity distribution of the HOMO and LUMO surfaces has been investigated, depicting much of the isodensities distribution on the heteratoms (50, 51).

#### Molecular electrostatic potential analysis

DFT studies were used to map another important electronic parameter, i.e., molecular electrostatic potential surfaces of Sil, Omtriptolid, Belamide A, Isomalyngamide A, and Daphnoretin, as indicated in **Figure 1 (III)**. It is evident from the figure that negative potenial is conventrated on oxygen, chlorine, and nitrogen atoms, which reflects elecron transfer from O, Cl, and S. Red color indicated that –O, –Cl, and –Scenters contribute as nucleophilic regions, whereas blue color indicates that –C, –N and –H atoms contribute as electrophilic regions.

#### 2D and 3D representation of TMZ– protein and Silymarin–protein complexes

**Figure 2:**
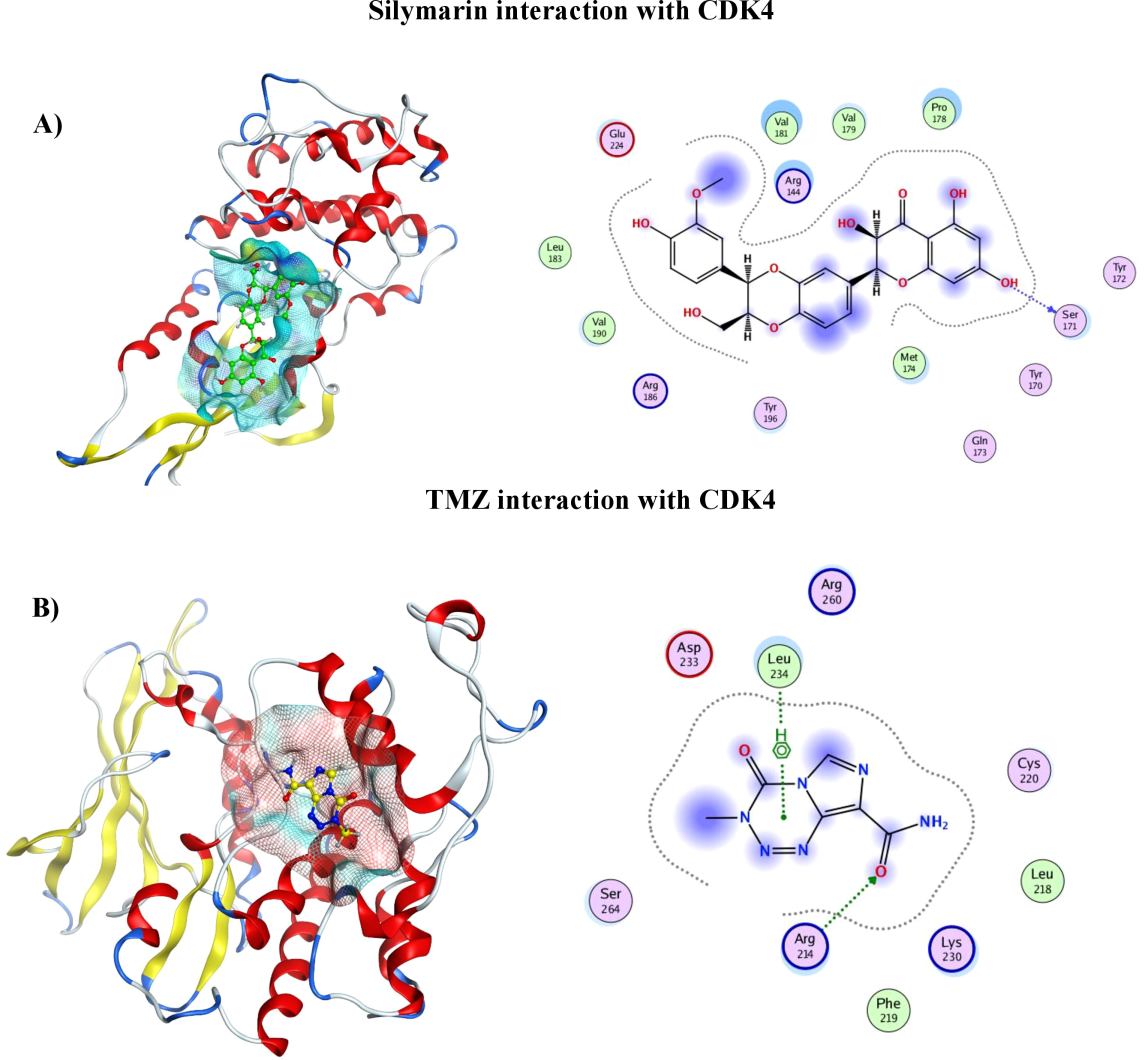
**(A)** 3D analysis of interaction of Silymarin in binding pocket of CDK4 (left), 2D LigPlot showing interactions of Silymarin with residues of protein (right). **(B)** 3D analysis of interaction of TMZ in binding pocket of CDK4 (left). 2D LigPlot showing interactions of Silymarin with residues of protein (right).

The protein–ligand interaction in 2D format shows that 14 amino acids of CDK4 interact with the Sil, as can be seen in **Figure 2 (I)**. Val181, Pro178, and Arg184 with half blue circle represent receptor exposure, whereas the ligand side chain with blue circle shows ligand exposure. The 2D depiction shows polar, lipophilic, acidic, and basic interaction between ligand and protein. In contrast, the TMZ interacts with nine amino acids, and the exposure of ligand–protein is less than that of Sil, as shown in the 2D structure represented in **Figure 2 (II)**. Drugs and protein exposure is shown by benzene ring (circled blue) and amino acids (colored blue). Amino acids that have a blue outline are basic in nature, whereas those having red outline are acidic.

### Characterization of SLNPs, SLNP-TMZ, and SLNP-Sil

#### SEM analysis

**Figure 3:**
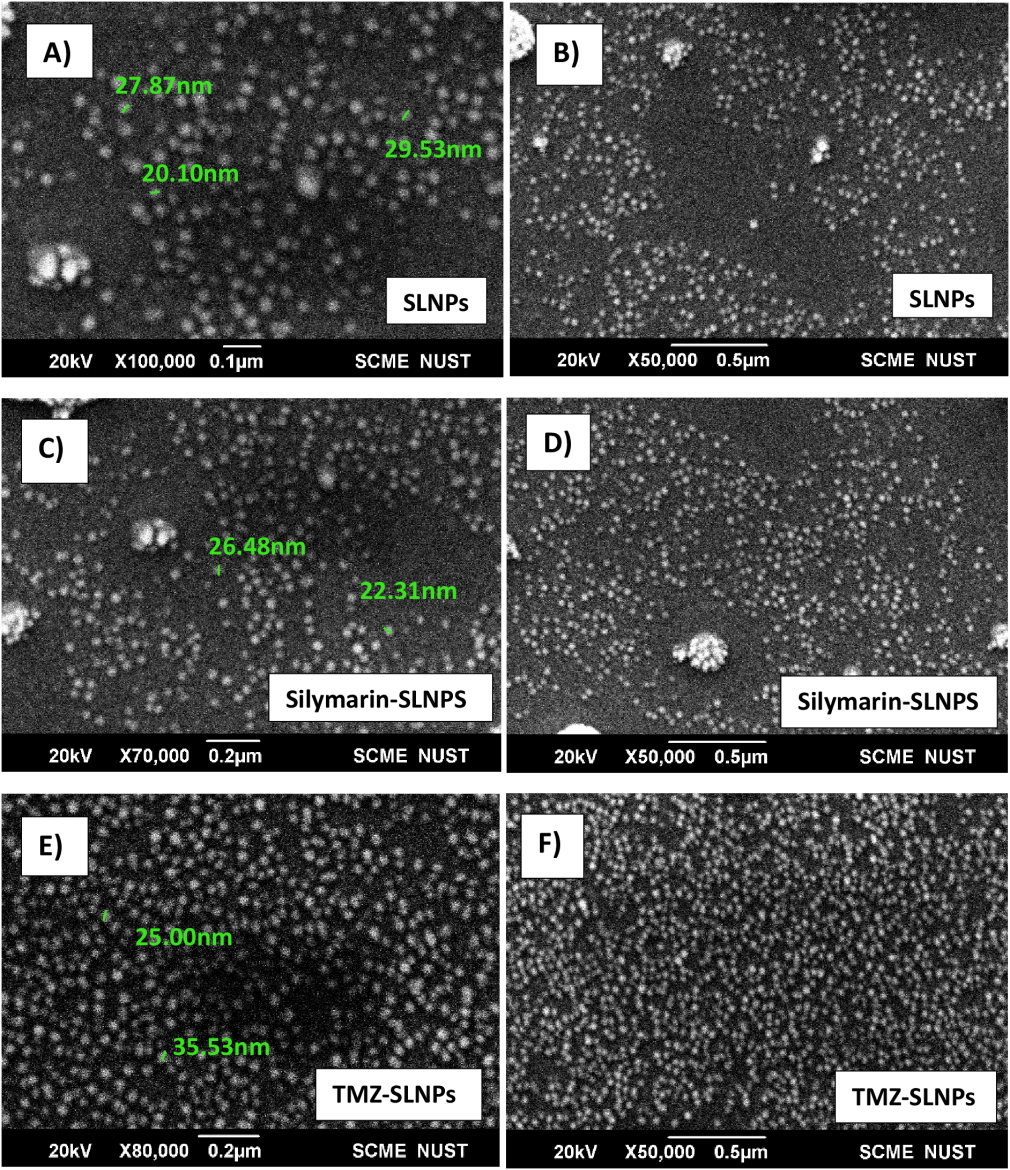
SEM images of blank solid lipid nanoparticles **(A, B)**, Silymarin- loaded solid lipid nanoparticles **(C, D)**, and TMZ- loaded Solid lipid nanoparticles **(E, F)** at ×100,000, ×80,000, and ×50,000 with size ranging from 20 nm to 35 nm.

To confirm the size and shape of blank SLNPs and SLNPs encapsulating Sil and TMZ, SEM analysis was performed. Images were taken at ×100,000, ×80,000, and ×50,000 magnifications with spatial resolution ranging from 0.1 µm to 0.5 µm. Images of the samples can be seen in **Figure 3**. From the images, the presence of SLNPs can be validated in spherical shape. Furthermore, the size range of SLNPs is confirmed for blank SLNPs, SLNP-Sil, and SLNP-TMZ, which ranges from 20 nm to 35 nm. This is an ideal size for any type of NPs to cross BBB.

#### FTIR analysis

**Figure 4:**
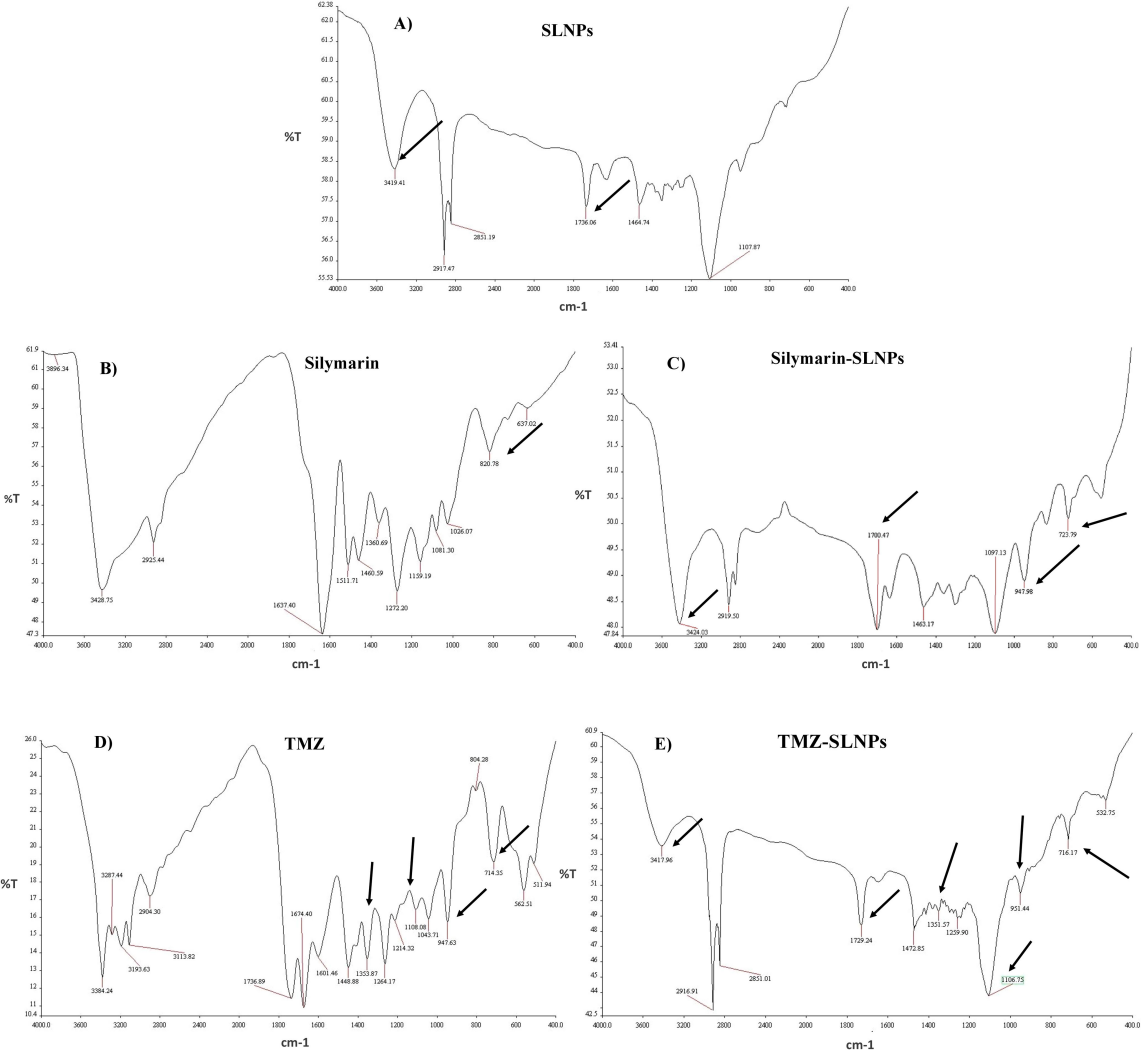
FTIR spectrum showing **(A)** blank solid lipid nanoparticles peaks, **(B)** Silymarin peaks, **(C)** Silymarin- encapsulated solid lipid nanoparticles peaks, **(D)** Temozolomide peaks, and **(E)** Temozolomide-encapsulated solid lipid nanoparticles. Characteristic peaks of Silymarin is shown on 3,419 cm^−1^, 1,736 cm^−1^, 947.98 cm^−1^, and 723.79 cm^−1^in SLNP-Sil, confirming Sil encapsulation in SLNPs. Characteristic peaks of TMZ is shown on 1,351.57 cm^−1^, 1,106.75 cm^−1^, 954.44 cm^−1^, and 716.17 cm^−1^in SLNP-TMZ, confirming TMZ encapsulation in SLNPs.

FTIR analysis for blank SLNPs, Sil, SLNP-Sil, TMZ, and SLNP-TMZ (**Figure 4**) was performed to validate and confirm the encapsulation of Sil and TMZ in SLNPs by comparing the characteristic peaks of pure drugs with the drug and NP conjugate. The spectrum analysis showed the presence of broad peak at 3,419 cm^−1^, which indicated the presence of O-H stretching, and the peak at 1,736 cm^−1^ indicated C = C group presence (36). The intensification of these peaks showed signs of Sil encapsulation. Characteristic peaks of Sil at 947.98 cm^−1^ indicating C=CH_2_ stretching and at 723.79 cm^−1^ indicating C–H bending vibrations and amine groups (–NH) further confirm Sil encapsulation in SLNPs (36, 52). Similarly, the FTIR peaks of TMZ- loaded SLNPs showed characteristic peaks of TMZ drug at 1,351.57 cm^−1^ indicating O–H bending, at 1,106.75 cm^−1^ indicating C–O–C stretching, at 954.44 cm^−1^ indicating C=CH_2_, and at 716.17 cm^−1^ indicating C–H bending vibrations and amine groups (–NH), which confirms TMZ encapsulation in SLNPs (53). Also, these drugs and SLNP complex showed no significant changes, indicating the absence of any chemical interaction between them and thus indicating that they were compatible with each other (53, 54).

#### XRD analysis

**Figure 5:**
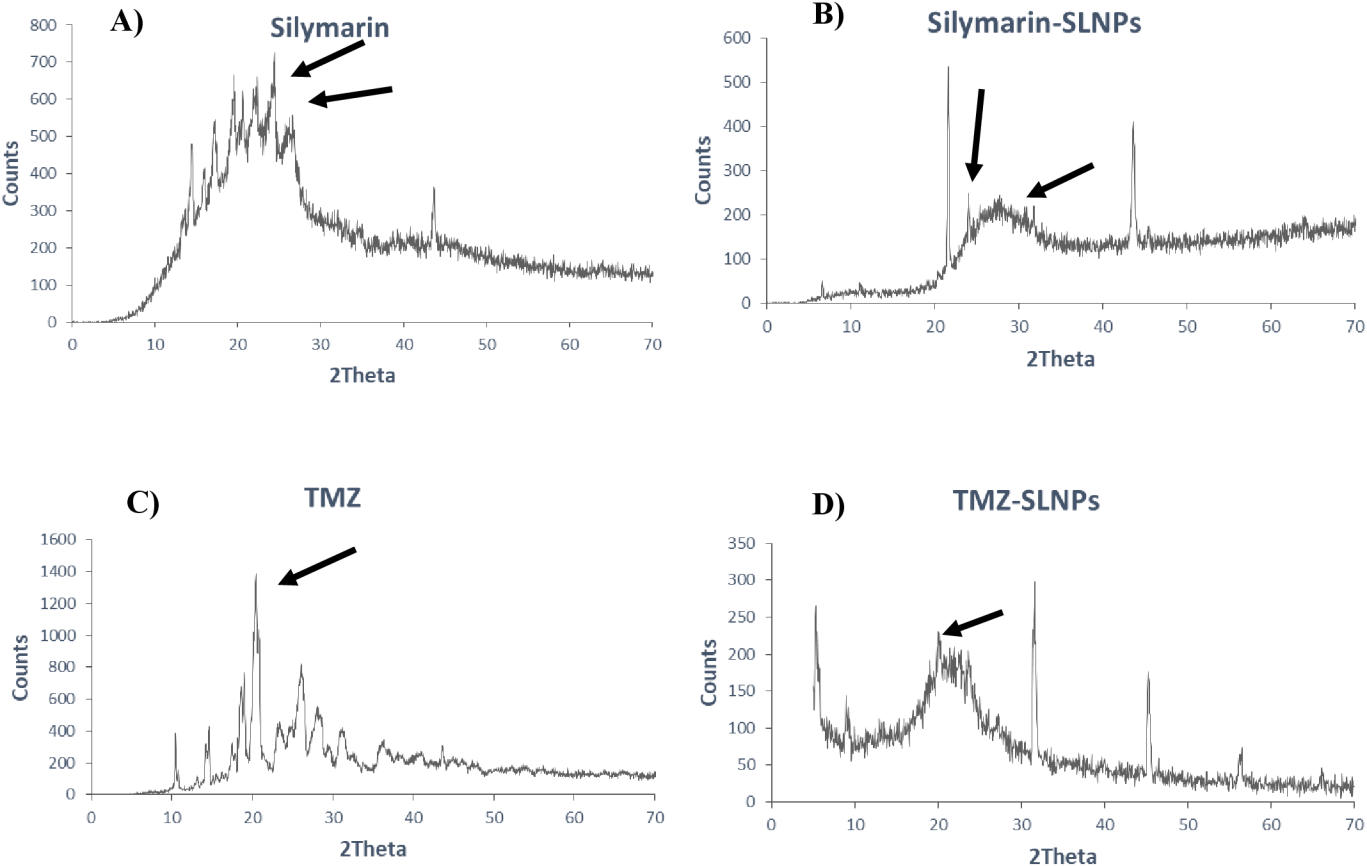
XRD patterns of **(A)** Silymarin, **(B)** Silymarin- encapsulated solid lipid nanoparticles, **(C)** Temozolomide, and **(D)** Temozolomide-encapsulated solid lipid nanoparticles. Both Silymarin and TMZ shows decrease in crystallinity when loaded in SLNP, as shown by reduction in crystalline peaks.

XRD was performed to identify the crystal state of TMZ, Sil, SLNP-TMZ, and SLNP-Sil. Patterns of blank SLNP, SLNP-TMZ, and SLNP-Sil were quite different from each other. Sil and TMZ powder showed a crystalline structure, as demonstrated by its sharp and intense diffraction peaks (**Figures 5A, C**), but, when loaded in SLNPs, the intensity of peaks was decreased, which indicates that, when Sil and TMZ are entrapped in SLNPs, its crystallinity decreases (**Figures 5B, D**). This less crystalline state of Sil and TMZ within SLNPs may contribute to increased drug solubility (55, 56).

### Drug entrapment and release efficiency

The entrapment efficiency of SLNPs for Sil was determined using UV/Vis spectroscopy. The equation of straight line obtained through calibration curve was y = 0.0039x + 0.4117, and R2 value was 0.9441. Results showed 81.5% entrapment efficiency of the SLNPS. Hence, excellent drug entrapment efficiency is found for SLNPs.

**Figure 6:**
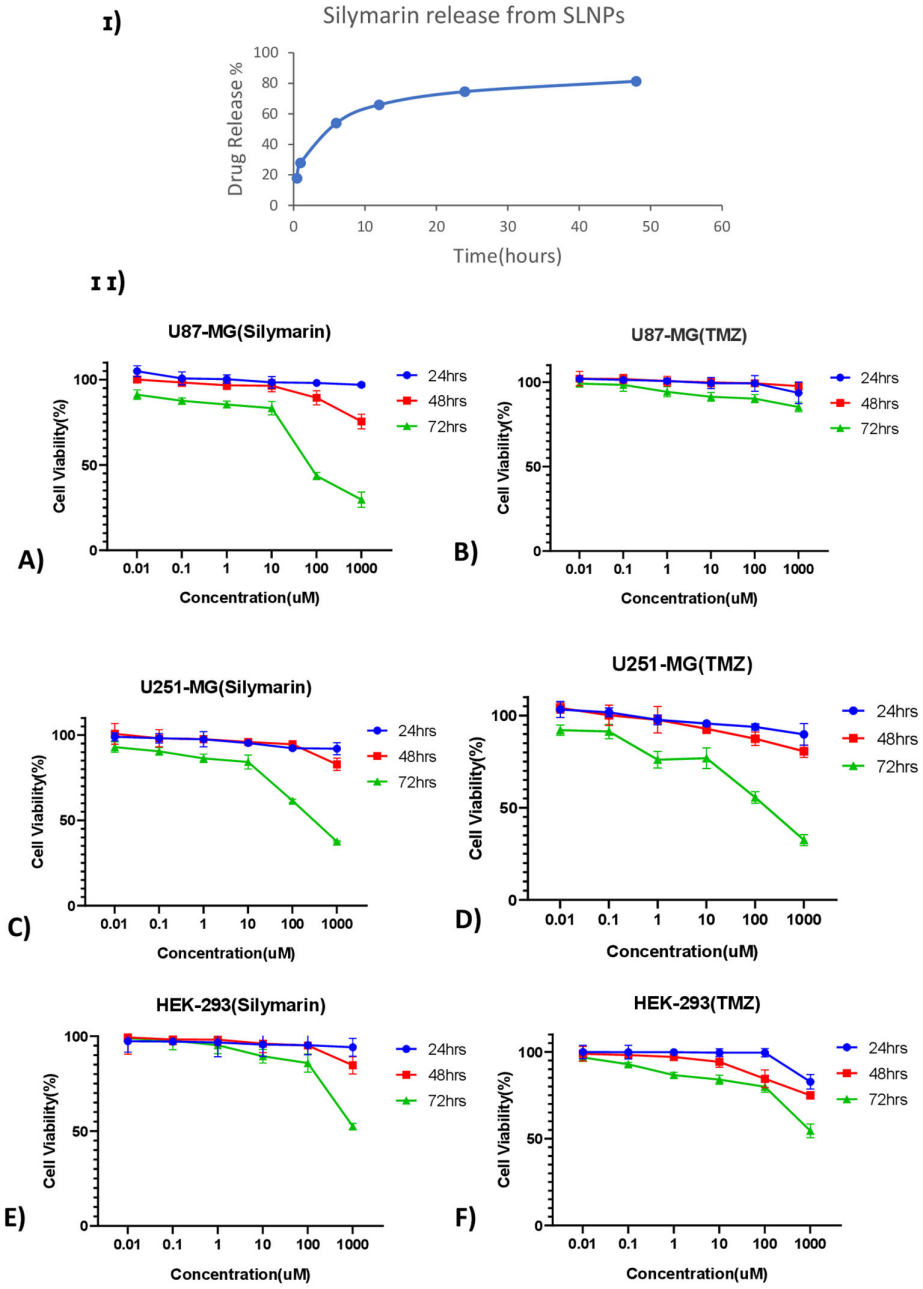
**(I)** Time- dependent Silymarin release from solid lipid nanoparticles observed at six different time points ranging from 30 min to 48 h and showing ~ 75% of drug release after 24 h **(II)** Line graphs show the cell viability of Silymarin and TMZ at different concentrations ranging from 0.01 µM to 1,000 µM at three different time points (24 h, 48 h, and 72 h) on **(A)** U87-MG, **(B)** U251-MG, and **(C)** HEK-293 cell lines.

Furthermore, the ability of SLNPs to release the drug was evaluated, and it was found that 17.8% of the drug is released in the solvent after 30 min, whereas 81.3% of the drug is released after 48 h [**Figure 6 (I)**]. Hence, a time -dependent release of SLNPs was observed.

### Anti-tumor activity

MTT assay was performed on U87-MG and U251-MG glioblastoma cell lines and HEK-293 to find out the cytotoxicity of Sil, TMZ, blank SLNPs, SLNP-Sil, and SLNP-TMZ (**Supplementary Data**). Initially, the effects of Sil, TMZ, and SLNPs were evaluated on all three cell lines for 24 h, 48 h, and 72 h at varying doses. A line graph of each cell line at each time point was plotted to compare the viability percentages of Sil, TMZ, and SLNPs. No considerable inhibitory effect of SLNPs, Sil, and TMZ was shown on glioblastoma cell lines as well as HEK-293 cell line after 24 h and 48 h of exposure. Activity of Sil, TMZ, and SLNPs were then observed after 72 h of treatment. Seventy-two hour s of treatment with Sil on U87 and U251 showed an obvious decrease in cell viability, and this cytotoxic effect was more obvious at higher concentrations. This cytotoxic effect was also shown by TMZ on U251, and this effect was almost equal to the effect shown by Sil on U251 at each concentration; however, on U87 cell line, TMZ showed no effect. Sil on HEK-293 after 72 h showed a negligible effect except at very high concentration, whereas TMZ had some cytotoxicity, but, compared to its cytotoxicity on U251, it was low. Seventy-two hours of treatment with SLNPs still had no considerable effect on glioblastoma cell lines and HEK-293. So, a concentration- and time-dependent inhibition of U87 and U251 cellular metabolic activity by Sil was confirmed [**Figures 6 (II) A, C**]. After treatment with 250-µM Sil for 72 h, more than 65% of the glioblastoma cells lost their viability.

### Statistical analysis

**Figure 7:**
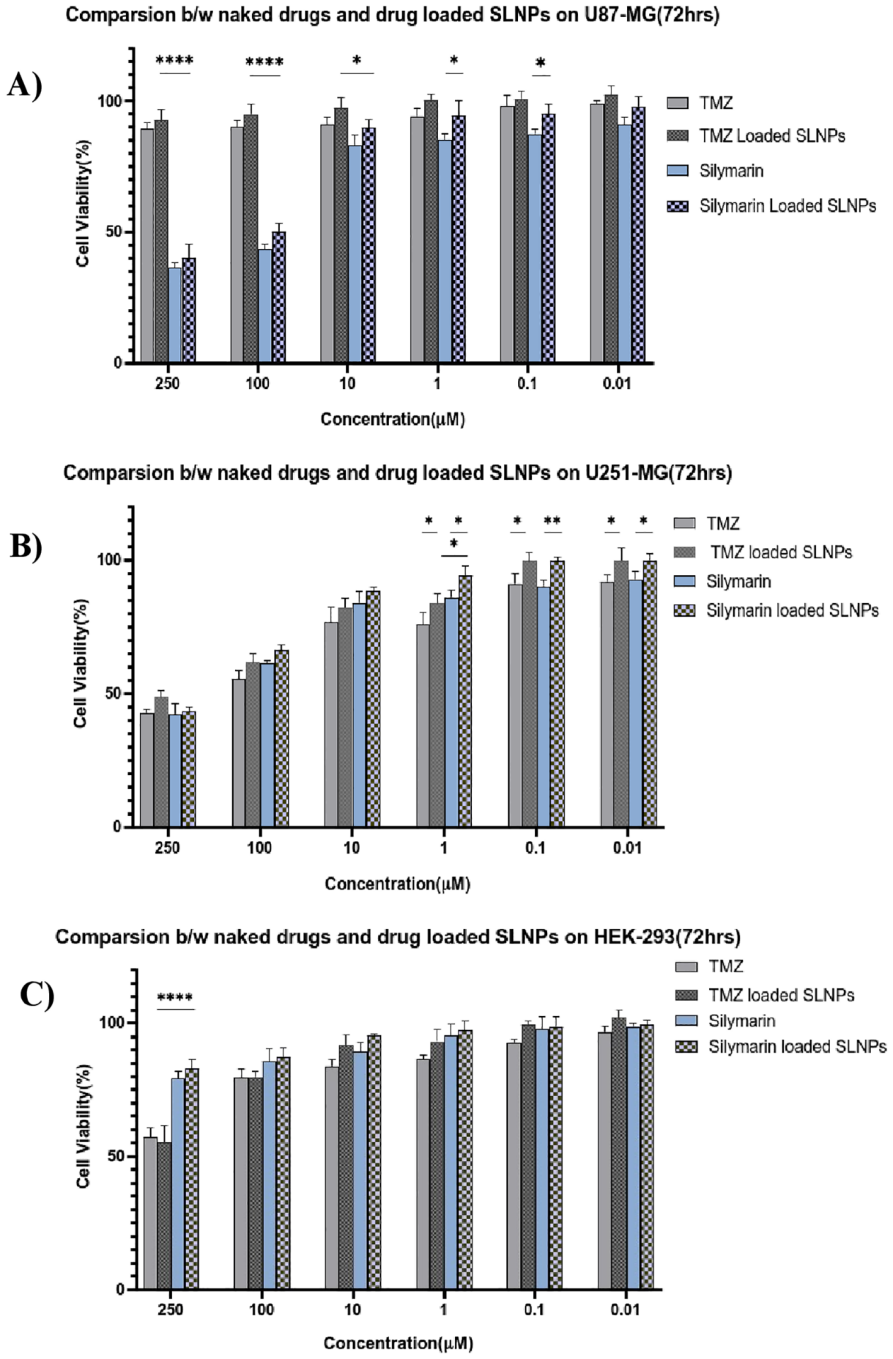
Bar graphs shows the comparison between naked Silymarin and Silymarin- encapsulated in SLNPs, aswell as naked TMZ and TMZ- encapsulated in SLNPs and at different concentrations and 72-h time point, on U87-MG **(A)**, U251-MG **(B)**, and HEK-293 **(C)** cell lines. Results have been presented as mean ± SEM. *p < 0.05, **p < 0.01, and ****p < 0.0001.

Sil and TMZ- loaded SLNPs cytotoxicity was also compared with naked drugs’ cytotoxicity at 72 h (**Figure 7**). MTT assay was performed with similar concentrations of naked and loaded drugs. The Sil and SLNP-Sil on U87, U251, and HEK did not differ significantly except at very low concentrations. Similarly, comparing TMZ and SLNP-TMZ, no significant difference was observed. However, when comparing Sil and TMZ- loaded in SLNPs, SLNP-Sil demonstrated significantly greater anti-tumor potential against U87 cancer cells, with an IC_50_ value of 155.14 µM, as compared to that of SLNP-TMZ (p < 0.001 and p < 0.01). In contrast, no significant difference was found between SLNP-Sil and SLNP-TMZ in U251 cancer cells (**Figure 7**).

### Impact of treatments on cell migration

**Figure 8:**
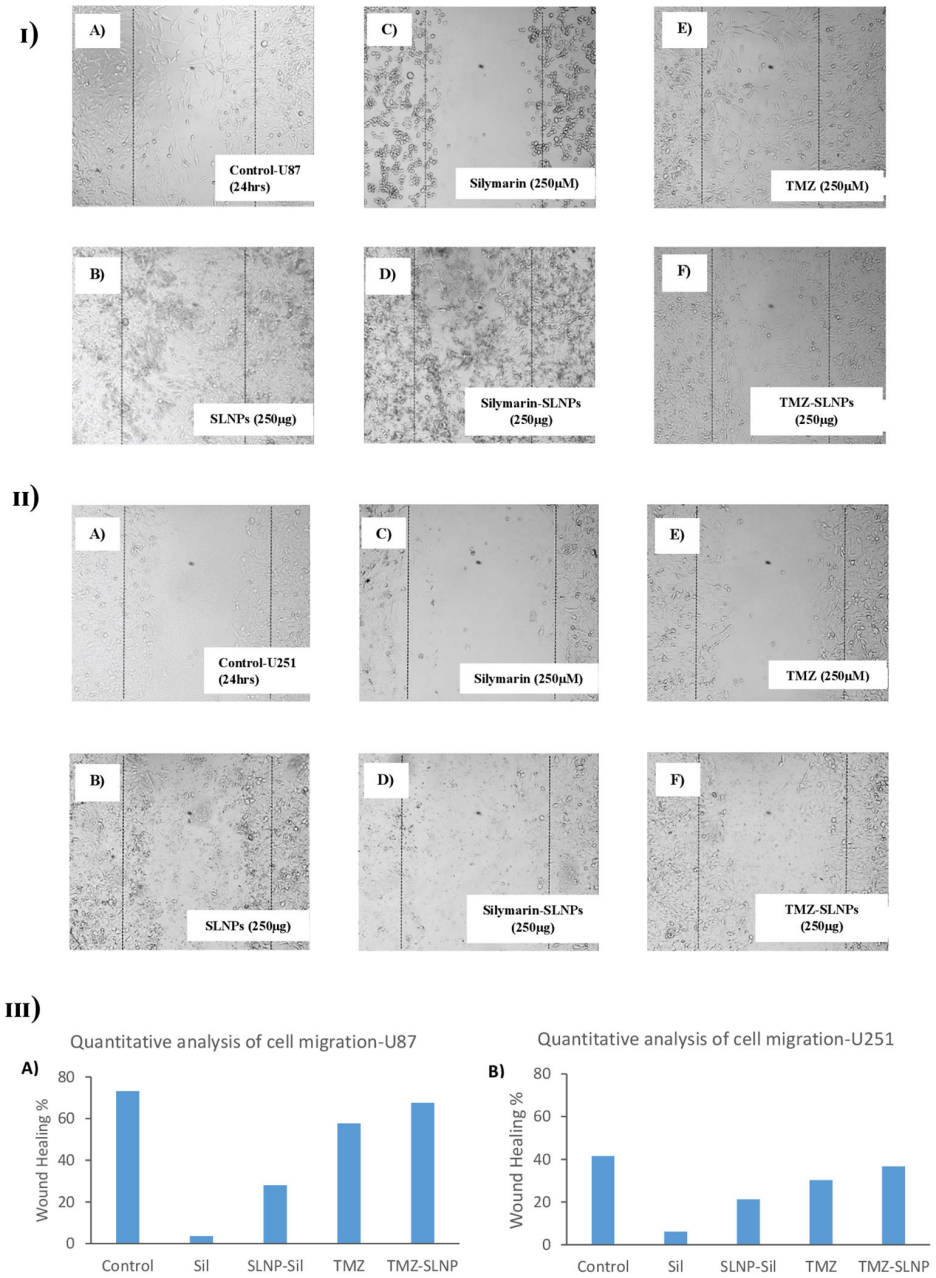
**(I)** Effect of **(A)** control **(B)** blank SLNPs **(C)** Silymarin, **(D)** SLNP-Sil, **(E)** TMZ, and **(F)** SLNP-TMZ on migration of U87 cells after 24 h. **(II)** Effect of **(A)** control **(B)** blank SLNPs **(C)** Silymarin, **(D)** SLNP- Sil, **(E)** TMZ, and **(F)** SLNP-TMZ on migration of U51 cells after 24 h. **(III)** Quantitative analysis of the percentage of wound healing in U87 **(A)** and U251 cells **(B)** after 24 h by untreated (control) and treated groups (Sil, TMZ, and their loaded nanoparticles). ImageJ software was used for this analysis. Migratory potential of both U87 and U251 cells is suppressed by Silymarin and Silymarin- loaded nanoparticles, whereas no hindrance in the migratory potential of the cells is observed when treated with naked SLNPs. Cells treated with TMZ and SLNP-TMZ showed very limited hindrance in cell migratory activities.

To investigate the inhibitory effect of Sil and SLNP-Sil on the tumor cells motility, cell migration assay was performed. Cells treated with bare SLNPs showed a significant potential to migrate, having no interruption in cell motility. Same results were noticed for control cells [**Figures 8 (I, II) A, B**]; however, cells treated with Sil and SLNP-Sil showed that they significantly suppressed the migration of U87 and u251 cells [**Figures 8 (I, II) C, D**]. This suppression in migration was seen to be concentration- dependent. Cells treated with TMZ and SLNP-TMZ showed very limited hindrance in cell migratory activities of U87 and U251 cells [**Figures 8 (I, II) E, F**].

## Discussion

For thousands of years, natural substances produced from animals, microorganisms, and, most notably, plants have been employed in the primary prevention and cure of human ailments. On a molecular level, compounds derived from plants have been shown to target a variety of biological activities. There are currently over 1,600 flavonoid-related patents and 3,000 polyphenol-related patents. It has been demonstrated that phytomolecules have pharmacological benefits for a range of disorders, including brain cancer.

Glioblastoma is the most aggressive form of brain cancer, with the lowest survival rate. Because of tumor heterogeneity, the chemotherapy employed to block tumor proliferation and spread is inadequate. Hence, chemotherapy resistance develops. In order to enhance the prognosis and survival rate of glioma patients, it is crucial to develop and identify drugs that can overcome resistance. This can be accomplished with medicinal plants. Medicinal plants contain a variety of phytochemicals that target aggressive brain tumors and trigger apoptosis, hence preventing glioma proliferation and recurrence (57–62). The current study aimed to evaluate therapeutic potential of Sil and Sil- encapsulated SLNPs against glioblastoma cell lines U87-MG and U251-MG.

A database of 691 plant extracts with potential anti-cancer, anti-proliferative, and anti-inflammatory properties reported in the literature have been generated in the first phase of the research. After identification of the compounds, they were docked with proteins of pRB pathway, which is altered in approximately 78% of glioblastomas. CDK2, CDK4, and CDK6 are the proteins that are targeted in the present study. CDK2 is an important cell cycle regulator that regulates cellular transitions from G1 to S phase and from G2 to M phase, whereas CDK4 and CDK6 govern G1- to -S phase transitions only. As a result, limiting the activation of these CDKs with inhibitors can successfully stop cancer cells from spreading by causing G1 or G2 cell cycle arrest (63). Similar study is documented where molecular docking is employed to identify novel inhibitors against target proteins of Streptococcus gallolyticus, which is a bactirum that causes infective endocarditis (inflammation of the heart lining) (64).

Molecular docking was performed to find the compounds with the best affinity for binding to the target proteins. The Lipinski rule was used to identify the plant extract that can act as a multi-target drug, targeting all the proteins listed above. However, none of the top compounds were found to act as a multi-target drug; therefore, the compounds targeting CDK4 only were further screened through DFT analysis after the Lipinski rule to identify the final compound that can be tested *in vitro* to validate this *in silico* study, which concluded that Sil is the final compound. CDK4 was focused as a target during *in silico* study because it is reported to be significantly more elevated in glioblastoma than CDK2 and CDK6.

Applying DSS approach, NPs were synthesized and characterized with the aim of passing the BBB. The BBB must be breached because it blocks the majority of medications from entering the brain (65). For this reason, SLNPs have been synthesized, which can act as a vehicle and efficiently carry medications to the brain while crossing the BBB (66). Several characterization methods, such as SEM, FTIR, and XRD analyses, were performed on both the blank and drug-loaded SLNPs following the synthesis of the SLNPs. Doxil, a liposome-encapsulated form of doxorubicin that enhances drug delivery to tumor cells while minimizing exposure to healthy tissues, particularly reducing cardiotoxicity, is approved by the FDA and has undergone extensive clinical trials demonstrating its efficacy and safety (67).

Particle size and shape were assessed through SEM, and the majority of the particles were found to be in the range of 10nm to 200 nm. Particle sizes exceeding 200 nm cannot cross BBB and accumulate in the liver and are eliminated from circulation by the complement system (36, 68). XRD analysis was performed to identify crystallinity of the drugs. XRD patterns of Sil marked its crystalline structure by showing diffraction peaks at different angles. However, the reduction in crystalline peaks of SLNP-Sil NPs revealed the successful encapsulation of the drug within SLNPs (69). FTIR analysis was performed to validate drug encapsulation in SLNPS. The distinctive Sil drug bands were visible in the FTIR peaks for SLNP-Sil, confirming that Sil is successfully loaded in SLNPs (**Figure 4**). Drug entrapment and release efficacy analysis of SLNPs indicated that, after 30 min, 17.8% of the drug was released into the solvent, whereas 81.3% of the drug was released and after 48 h. The results indicated that SLNPs exhibited time-dependent drug release. This demonstrates that, as stated by prior research, these NPs have a greater surface area for drug trapping due to their small size (9).

Cytotoxic effect of Sil, TMZ, and their loaded SLNPs against U87, U251 glioma cell lines, and HEK-293cell line in an *in vitro assay* revealed significant conclusions. Sil and SLNP-Sil show dose-dependent and time-dependent cytotoxicity against glioblastoma cell lines. At 72 h, Sil exhibited cytotoxicity at concentration as low as 0.01 M, and there was no significant difference between the cytotoxic potential of Sil and SLNP-Sil (70). This demonstrated that Sil retained its potent cytotoxic effect against glioma cell lines when encapsulated in SLNPs (36), whereas it mildly affects the proliferation of the normal cell line (HEK-293). IC_50_ value of Sil came out to be 155.14 µM for U87 and 195.93 µM for U251. The TMZ drug was subsequently analyzed in this manner. On U87, negligible cytotoxicity was observed, whereas TMZ exhibited cytotoxicity on U251, at 72 h, which was comparable to the cytotoxicity exhibited by Sil on this cell line (71, 72). IC_50_ value of TMZ came out to be 1177.019 µM for U87 and 172.4574 µM for U251 cell line.

Through a cell migration assay, the effect of SLNPs, Sil, TMZ, and their loaded NPs on the migratory potential of U87 and U251 cells was determined. Results demonstrated that Sil and SLNP-Sil prevented U87 and U251 cells from migrating. However, cells treated with bare SLNPs demonstrated a significant capacity for migration with no disruption of cell motility. Additionally, TMZ and TMZ- loaded SLNPs only mildly hampered the migration of U87 and U251 cells.

This study demonstrates that the BBB can be overcome by formulating NPs, capable of encapsulating a drug and transporting it to the target site. Small size, lower toxicity, greater stability, and biocompatibility make SLNPs efficient drug delivery vehicle across the BBB. *In silico* analysis predicted Sil as the most potent anti-cancer and anti-proliferative drug against CDK4, altered in GBM. Sil- loaded SLNPs through *in vitro* experimentations proved to be efficient to control cancer cell proliferation.

While our study provides promising insights into the cytotoxic and anti-migratory effects of Sil and its SLNP formulation, it is important to note the limitations. This study did not directly investigate the molecular mechanisms through which Sil exerts its anti-cancer effects, particularly its impact on the CDK4/pRB pathway, rather it provides an insight into the Sil and SLNP formulation ability to show its anti-proliferative and anti-cancerous effect on the cell line. This research in identifying and characterizing a novel GBM treatment approach is yet to be analyzed more in depth. Other aspects of anti-cancer activity such as the formulation’s effects on apoptosis, cell cycle arrest, and interaction with the tumor microenvironment remain to be investigated. Further experimentations are needed to fully understand its therapeutic potential, including *in vivo* efficacy, pharmacokinetics, biodistribution, long-term safety, and optimal dosing strategies. Specifically, pathway-specific assays are required to confirm CDK4 inhibition and its downstream effects on the pRB pathway. Recent studies have highlighted the importance of conducting kinase activity assays, flow cytometry for cell cycle analysis, and Western blotting for the phosphorylation status of pRB to validate whether compounds effectively inhibit CDK4-mediated signaling. In conclusion, while this study provides a promising start for the development of a new glioblastoma treatment, it represents the beginning rather than the completion of the research journey. These findings lead us to further investigate the development of a clinically viable treatment. The next steps will focus on the *in vivo* validation, detailed mechanistic studies, and exploring the potential for combination therapies to maximize therapeutic efficacy.

## Data Availability

The raw data supporting the conclusions of this article will be made available by the authors, without undue reservation.
