# Supplementary material for: SLNP-based CDK4- targeted nanotherapy against glioblastoma
Source: Front Oncol. 2024 Nov 22;14:1455816. doi: 10.3389/fonc.2024.1455816 (PMC11621005; doi:10.3389/fonc.2024.1455816)
Supplement: Supplementary file 1 [file DataSheet1.docx]

Figure. Calibration curve of Silymarin
